# Gene Expression-Based Dosimetry by Dose and Time in Mice Following Acute Radiation Exposure

**DOI:** 10.1371/journal.pone.0083390

**Published:** 2013-12-16

**Authors:** James D. Tucker, George W. Divine, William E. Grever, Robert A. Thomas, Michael C. Joiner, Joseph M. Smolinski, Gregory W. Auner

**Affiliations:** 1 Department of Biological Sciences, Wayne State University, Detroit, Michigan, United States of America; 2 Department of Public Health Sciences, Henry Ford Hospital, Detroit, Michigan, United States of America; 3 Department of Radiation Oncology, Wayne State University, Detroit, Michigan, United States of America; 4 Department of Electrical and Computer Engineering, Wayne State University, Detroit, Michigan, United States of America; ENEA, Italy

## Abstract

Rapid and reliable methods for performing biological dosimetry are of paramount importance in the event of a large-scale nuclear event. Traditional dosimetry approaches lack the requisite rapid assessment capability, ease of use, portability and low cost, which are factors needed for triaging a large number of victims. Here we describe the results of experiments in which mice were acutely exposed to ^60^Co gamma rays at doses of 0 (control) to 10 Gy. Blood was obtained from irradiated mice 0.5, 1, 2, 3, 5, and 7 days after exposure. mRNA expression levels of 106 selected genes were obtained by reverse-transcription real time PCR. Stepwise regression of dose received against individual gene transcript expression levels provided optimal dosimetry at each time point. The results indicate that only 4–7 different gene transcripts are needed to explain ≥ 0.69 of the variance (R^2^), and that receiver-operator characteristics, a measure of sensitivity and specificity, of ≥ 0.93 for these statistical models were achieved at each time point. These models provide an excellent description of the relationship between the actual and predicted doses up to 6 Gy. At doses of 8 and 10 Gy there appears to be saturation of the radiation-response signals with a corresponding diminution of accuracy. These results suggest that similar analyses in humans may be advantageous for use in a field-portable device designed to assess exposures in mass casualty situations.

## Introduction

In situations where large numbers of people may be exposed to ionizing radiation, a rapid, sensitive and reliable means of performing biological dosimetry is critical for triage and initial clinical assessment. To this end the feasibility of using PCR-based gene expression analyses for dosimetry was evaluated. Blood was obtained from C57BL/6 male mice acutely exposed to 0 to 10 Gy of whole-body ^60^Co gamma rays and evaluated at times ranging from 0.5 to 7 days following exposure.

Animal models offer biodosimetry information in a more controlled system than is possible with humans. The mouse is commonly used as a model organism to evaluate analytical approaches for assessing exposures to ionizing radiation. The use of animals is essential because in humans, unintended whole body exposures are rare, the doses received are usually unknown and cannot be reliably estimated in a timely manner, and the exposures may be partial body or nonuniform. Dose rates may differ among recipients and may not be known. Radiation exposures may also be accompanied by burns, chemical exposures, and physical trauma such as broken bones and even imbedded debris. Post-event assessment of radiation exposures may be further influenced by pre-existing lifestyle choices including cigarette smoking and factors not under personal control such as age and genotype. Assessments may also be influenced by the amount of time elapsed after exposure since many biomolecular signals change over time. Some of these variables may have substantial interactions with each other.

 Commonly used methods of radiation biodosimetry include cytogenetic evaluations of structural chromosome aberrations, e.g. [1–5] which have been the gold standard for many years but these assays are costly and can take weeks to conduct. Gene mutation assays such as HPRT [1,6,7] and Glycophorin A [1,6,8,9] have also been used, although these are not usually as accurate as cytogenetics [1] and only work on individuals of the appropriate genotype or gender. Electron paramagnetic resonance has also been used successfully [9]. Until recently [10,11] this approach generally required relinquishing a tooth for analysis, which precluded widespread application of this method. However, none of these methods is suitable for evaluations leading to triage in mass casualty situations because they are either too expensive, too slow to perform, require highly skilled laboratory personnel, or because they require samples to be sent to an analytical laboratory rather than having the analytical capability transported to the triage location.

Here we used reverse-transcription real time PCR (qPCR) to quantify the expression of selected genes as a function of time out to 7 days post-exposure. Throughout this entire assessment period many of the gene transcripts showed promise for use in dosimetry, while others were useful as endogenous controls. We conclude that gene expression analyses by qPCR shows considerable promise as a rapid method for performing radiation biological dosimetry.

## Results

Table 1 shows the number of mice evaluated for gene expression at each dose and time point. For many treatment groups the number of mice irradiated was larger than the number of animals evaluated for gene expression. Mice were most likely to be lost to analysis if they received a dose of 4 Gy or higher, and two or more days had elapsed since exposure. The most common reasons for unusable data values were low RNA yield or invalid PCR reactions.

**Table 1:** Number of mice evaluated by dose and time.

	**Days post-irradiation**						
**Dose (Gy)**	**0.5**	**1**	**2**	**3**	**5**	**7**	**Day total**
Naïve	24	30	26	35	42	38	195
Sham	20	33	27	47	30	38	195
0.5	12	16	19	20	21	19	107
1	15	23	19	21	14	19	111
1.5	20	13	14	18	18	18	101
2	20	35	42	31	41	30	199
2.5	17	16	15	14	14	18	94
3	18	39	23	33	36	34	183
4	21	44	21	28	31	36	181
6	15	45	34	28	30	26	178
8	16	45	33	23	37	29	183
10	13	21	15	11	13	11	84
Dose total	211	360	288	309	327	316	1811

**Figure 1:**
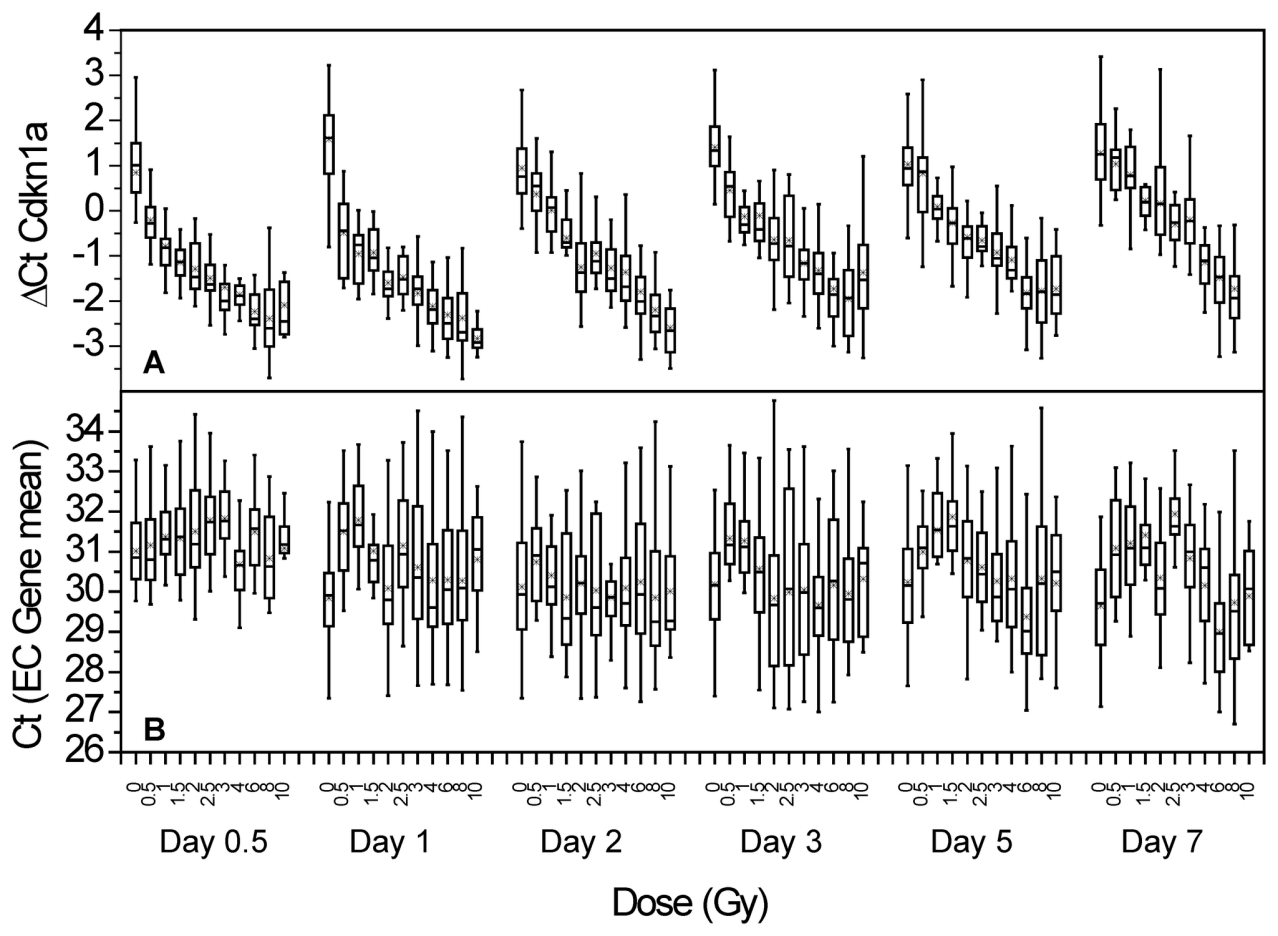
Cycle threshold (Ct) values measured by qPCR. Cdkn1a is shown in the top panel, and the set of 3 endogenous control (EC) gene transcripts are shown in the lower panel. The declining Cdkn1a responses indicate a significant dose-responsive increase in mRNA levels which persist for 7 days. These results are in contrast to the EC gene responses which are comparatively flat and show no systematic change with dose or time.

 The selected set of endogenous control (EC) genes is listed in Table 2. The average Ct expression values for EC genes are shown in Figure 1. These values are remarkably consistent over time and across all 11 dose groups. The ΔCt values for the most commonly observed response gene, Cdkn1a, are also shown in Figure 1. Here the ΔCt values for each time point decline markedly as dose increases, indicating not only a large increase in expression levels with dose, but strong persistence over time since exposure.

**Table 2:** M-values and statistics for endogenous control genes.

**Probe**	**M-Value**	**% of samples yielding valid data**	**Std. Dev. of the average Ct value**
Erp44-Mm00466483_m1	0.630	99.0%	1.56
Gtf3a-Mm00550608_m1	0.651	98.6%	1.58
Tmem168-Mm00551402_m1	0.656	98.5%	1.56

**Figure 2:**
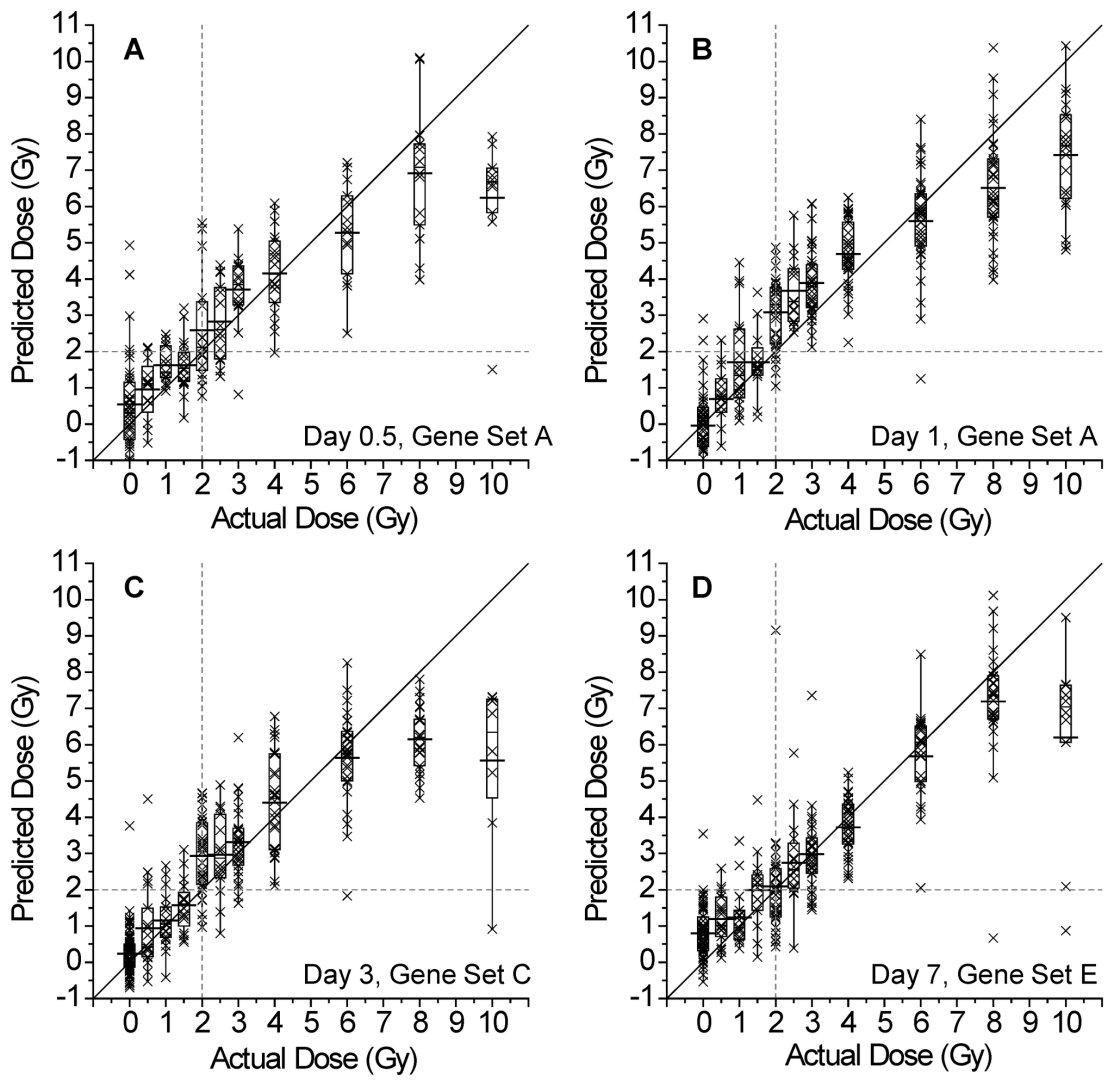
Relationship between the actual dose administered and the predicted dose. Panels A and B: Gene Set A, used in both these models, is described in Table 3. Panels C and D: Gene Sets C and E were used in these models and are described in Tables 5 and 7, respectively. The observations for each dose are summarized by box plots, as well as an X for each individual animal. The vertical and horizontal dashed lines at 2 Gy illustrate how many animals would have their exposure estimated correctly (upper right and lower left quadrants in each image as defined by these lines) and incorrectly (false positives in the upper left and false negatives in the lower right quadrants) with respect to an exposure of 2 Gy or higher. The diagonal line indicates where each measured value would lie if the predicted dose was the same as the actual dose.

The predicted radiation dose estimates derived from the selected multiple regression models are shown in Figure 2. The estimates for all the other time points are shown in Figures S1 and S2. The predicted radiation doses for each time point provide a very close fit to actual radiation doses up to 6 Gy. However, at 8 Gy and especially at 10 Gy the predicted dose estimates are lower than the actual doses, indicating that gene expression levels in mice receiving more than 6 Gy are lower than expected which shows that the linear relationship between gene expression and dose does not hold at these higher doses.

**Figure 3:**
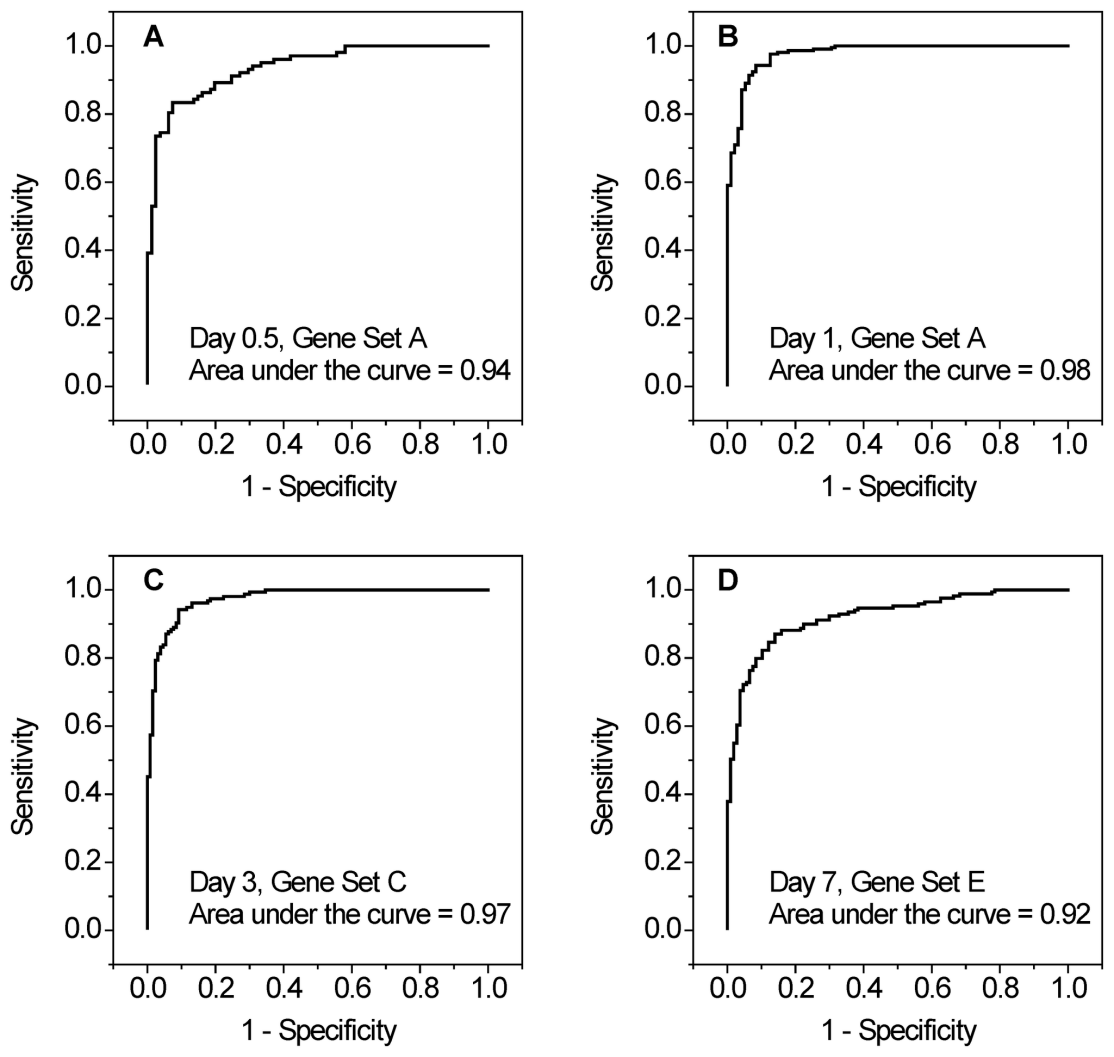
Receiver-operator characteristic curves. Large areas under the curve, as shown here, indicate high levels of sensitivity and specificity of the models. These curves correspond to the models in Figure 2.

While our dosimetry models provide accurate exposure predictions based on gene expression at doses up to 6 Gy, a major goal of this study was to determine the feasibility of using gene expression to predict exposures above or below a pre-determined threshold value. This ability is of critical importance in triage settings in the wake of a nuclear event. For this study a threshold value of 2 Gy was used, thus we employed a concentration of doses around this value. The ability of each dosimetry model to predict exposures above or below 2 Gy was assessed by evaluating its receiver-operator characteristic (ROC) curve in which the sensitivity is plotted versus 1 minus the specificity. (The sensitivity is the fraction that is true positive out of the total number truly exposed to 2 Gy or above, and 1-specificity is the fraction that is false positive out of the total number truly exposed to below 2 Gy.) Representative ROC curves corresponding to the time points in Figure 2 are provided in Figure 3; all the remaining ROC curves are shown in Figures S3 and S4. These curves indicate that each model provides a high level of both sensitivity and specificity. An ideal classification would yield a ROC curve with an area under the curve (AUC) of 1.0, while an AUC of 0.5 represents a random classification. For the ROC curves generated by our dosimetry models, AUCs of 0.92 to 0.98 were obtained, indicating that, for our data, the model has a high level of ability to predict exposure at a threshold of 2 Gy. From Figures 2 and 3 it is evident that the statistical models provide an excellent description of the relationship between the actual and predicted doses up to 6 Gy. However, it is worth noting that while saturation diminishes the ability to perform dosimetry above 6 Gy, the measured response is highly discernible from doses below 6 Gy.

Tables 3-7 list the gene transcripts for the 5 sets of genes (A, B, C, D, E, where each set corresponds to a pair of adjacent time points), that were found to contribute significantly to the dosimetry models. Even though the models contain only 4 to 7 gene transcripts, they explain 0.69 to 0.80 of the total variance, and are especially accurate at doses of 6 Gy and lower. Cdkn1a appears first on each list in Tables 3-7, indicating that expression of this mRNA is significantly elevated throughout the first week following exposure. Cdkn1a consistently explained more than half the total variation, clearly indicating that this gene is the single best predictor of ionizing radiation exposure under the conditions evaluated here. The retinoblastoma (Rb1) mRNA appears at every time point except day 5 (model D), indicating that this gene product is also a statistically significant predictor of exposure. At each time point there were also several additional gene transcripts that met the criteria for inclusion in these models, and although their contributions were statistically significant, none appeared as consistently as Cdkn1a.

**Table 3:** Stepwise variable selection summary for days 0.5 and 1 (Gene Set A).

		**Day 0.5 (N=211)**			**Day 1 (N=351)**		
**Step**	**(New) Variable(s)**	**R^2^**	**N (% Max)**		**R^2^**	**N (% Max)**	
1	Cdkn1a, Cdkn1a_sq	0.509		203 (96.2%)	0.579		337 (96.0%)
2	Plxnb2, Plxnb2_sq	0.615		193 (91.5%)	0.616		332 (94.6%)
3	Rb1	0.635		192 (91.0%)	0.638		330 (94.0%)
4	Tlk1, Tlk1_sq	0.650		190 (90.0%)	0.681		329 (93.7%)
5	Hmbs	0.668		187 (88.6%)	0.703		317 (90.3%)
6	Hdac2	0.678		186 (88.2%)	0.717		313 (89.2%)
7	Stom	0.688		183 (86.7%)	0.744		305 (86.9%)

**Table 4:** Stepwise variable selection summary for days 1 and 2 (Gene Set B).

		**Day 1 (N=351)**			**Day 2 (N=284)**		
**Step**	**(New) Variable(s)**	**R^2^**	**N (% Max)**		**R^2^**	**N (% Max)**	
1	Cdkn1a, Cdkn1a_sq	0.579		337 (96.0%)	0.615		272 (95.8%)
2	Rb1, Rb1_sq	0.618		335 (95.4%)	0.704		270 (95.1%)
3	Tlk1	0.660		334 (95.2%)	0.745		263 (92.6%)
4	Fuca1, Fuca1_sq	0.670		333 (94.9%)	0.765		263 (92.6%)
5	Ccng1, Ccng1_sq	0.693		329 (93.7%)	0.784		256 (90.1%)
6	Prkdc	0.721		302 (86.0%)	0.800		249 (87.7%)

**Table 5:** Stepwise variable selection summary for days 2 and 3 (Gene Set C).

		**Day 2 (N=284)**			**Day 3 (N=306)**		
**Step**	**(New) Variable(s)**	**R^2^**	**N (% Max)**		**R^2^**	**N (% Max)**	
1	Cdkn1a, Cdkn1a_sq	0.615		272 (95.8%)	0.536		295 (96.4%)
2	Rb1, Rb1_sq	0.704		270 (95.1%)	0.669		293 (95.8%)
3	Tlk1	0.745		263 (92.6%)	0.683		289 (94.4%)
4	Ccng1, Ccng1_sq	0.773		256 (90.1%)	0.699		287 (93.8%)
5	B2m	0.784		254 (89.4%)	0.725		285 (93.1%)

**Table 6:** Stepwise variable selection summary for days 3 and 5 (Gene Set D).

		**Day 3 (N=306)**			**Day 5 (N=324)**		
**Step**	**(New) Variable(s)**	**R^2^**	**N (% Max)**		**R^2^**	**N (% Max)**	
1	Cdkn1a, Cdkn1a_sq	0.536		295 (96.4%)	0.550		315 (97.2%)
2	Ccna2, Ccna2_sq	0.686		287 (93.8%)	0.728		309 (95.4%)
3	Ccng1, Ccng1_sq	0.703		285 (93.1%)	0.744		307 (94.8%)
4	Actb	0.786		264 (86.3%)	0.785		285 (88.0%)

**Table 7:** Stepwise variable selection summary for days 5 and 7 (Gene Set E).

			**Day 5 (N=324)**			**Day 7 (N=316)**		
**Step**	**(New) Variable(s)**		**R^2^**	**N (% Max)**		**R^2^**	**N (% Max)**	
1		Cdkn1a, Cdkn1a_sq	0.550		315 (97.2%)	0.534		296 (93.7%)
2		Rb1, Rb1_sq	0.710		314 (96.9%)	0.633		293 (92.7%)
3		Ticam2	0.767		295 (91.0%)	0.701		287 (90.8%)
4		Ssr1, Ssr1_sq	0.781		283 (87.3%)	0.724		276 (87.3%)

 To confirm these models we performed cross-validation analyses, which are summarized in Tables S1, S2, S3, and S4 in File S1. Although Cdkn1a was included in every validation model, and was the first variable chosen in all but one instance, the other variables selected using each half of the sample varied. In the validation models the R-square statistics dropped by an average of 0.127. The area under the ROC curves were more stable, going down an average of 0.02, and increased in 5 of the 20 instances.

## Discussion

 We have shown in mice that mRNA levels from just 4-7 genes explain at least 0.69 of the variance following a single acute exposure to ^60^Co for doses up to 6 Gy. The sensitivity and specificity of these models is at least 0.93 for each time point out to 7 days post-exposure, and that dosimetry is retained for this entire 7-day period. These models provide an alternative to the microarray-based approach for determining radiation exposure both *ex vivo* and *in vivo* which has similarly demonstrated a high degree of sensitivity and specificity in radiation dose prediction (e.g. 12). In the microarray approaches, as many 74 gene transcripts may be needed for dosimetry, at least in humans, but arguably with a simpler quality control. By comparison, the qPCR approach we report here requires analysis of considerably fewer genes but with better quality control required to maintain reproducibility and accuracy.

 Our finding that a modest number of gene transcripts is sufficient for dosimetry may simplify the task of dose estimation in humans following a large-scale radiological incident. Fewer gene transcripts means fewer analyses to run, reducing costs and possibly decreasing the read-out time. Our choice to use PCR to quantify gene expression allowed us to see changes in expression levels as large as 30-fold, which is much greater than can be achieved by microarray analysis. This dynamic range enables increased sensitivity to changes in gene expression levels, which means smaller blood volumes can be evaluated. These small volumes could be achieved by tail nicking in the case of rodent-based research, or in humans by a finger-stick and a capillary tube rather than the more routine phlebotomy.

 Although individual mRNA molecules are well-known to have short half-lives, our results show that at least some genes maintain their radiation-induced altered expression levels for extended periods of time. This long-term persistence of radiation-induced changes in expression bodes well for the possibility of performing dosimetry in humans many days after exposure. Numerous circumstances may prevent dosimetry from being performed shortly after exposure. In the case of a large event, e.g., Fukushima [13], Chernobyl [14], or a “dirty bomb”, there may be infrastructure damage and power outages as well as wide-spread panic that inhibits emergency personnel from getting to where they are needed. The ability to perform dosimetry many days after an event could be a distinct advantage under such circumstances.

Cdkn1a was the best predictor of radiation exposure at each time point (Tables 3–7), an observation that was strongly supported by the validation modeling (Tables S1, S2, S3, and S4 in File S1). Interestingly, Cdkn1a is also sensitive to radiation exposure in humans, e.g., [15], including low doses. In the work described here Cdkn1a is responsive at high doses, and explains at least 0.50 of the total variance at each time point in our multiple regression models. Combined, the remaining genes in these models for each time point explained no more than 0.25 of the variance, so Cdkn1a alone is clearly the primary variable explaining the radiation responses.

We have demonstrated in mice that qPCR can reliably differentiate between whole body doses above and below 2 Gy. In humans, this is a dose below which critical symptoms of radiation exposure are unlikely, and where clinical intervention is unnecessary. The acute whole-body dose for 0.50 lethality in humans is approximately 4.5 Gy [16]. In C57BL/6 mice at the age used in these studies, a higher LD_50_ of approximately 6.1 Gy has been reported [17]. It is not yet known if the different LD_50_ would reflect a different extent of gene expression for a given radiation dose in mice compared with humans as measured by qPCR. However, microarray studies indicate that similar gene expression changes in humans are seen over the range of doses we report here for qPCR in mice [12,15]. Since the dose range of expression measured by qPCR is greater than can be achieved by microarray analysis, it is therefore likely that qPCR will be at least as sensitive in differentiating human radiation exposure above and below 2 Gy as we have shown in mice. 

The results reported here were obtained with a single strain of fully-inbred male mice of uniform age. This contrasts the situation with humans where gender, age, lifestyle, and other exposures are potential confounders. Nevertheless, this work provides an important proof-of-concept that gene expression changes can persist for at least a week following acute whole-body radiation exposure in a mammalian model.

In summary, excellent radiation dosimetry is attained by using qPCR to evaluate expression levels of genes for at least 7 days after an acute dose of ionizing radiation. Dosimetry up to 6 Gy is very accurate. Above 6 Gy we appear to be seeing the limits of gene expression for dosimetry, at least in mice. Whether the same is true in humans is not clear and needs to be investigated. Extension of the approach described here for assessing adverse exposure to humans appears feasible and bodes well for a small, field-portable device for radiation dosimetry following a radiological incident.

## Materials and Methods

### Ethics Statement

This use of animals was carried out in strict accordance with the recommendations in the Guide for the Care and Use of Laboratory Animals of the National Institutes of Health. The protocol was approved by Wayne State University’s Institutional Animal Care and Use Committee (Protocol A 11-03-09). All cardiac punctures were performed under CO_2_-induced narcosis.

### Animal husbandry

Male C57BL/6 mice (Harlan Laboratories) 10-12 weeks old were used. After shipping, mice were acclimated for at least six days before experimental manipulation. 

### Radiation exposures and dosimetry

All radiation exposures were given with a clinical ^60^Co Theratron teletherapy unit (MDS Nordion, Ottawa, Canada). For irradiation, mice were placed into circular, clear plastic “pie cages” (Braintree Scientific, Inc., Braintree, MA). Each pie cage held 10 mice in individually separated wedge-shaped compartments of size 5 x 9 cm, allowing freedom of movement. Animals were not sedated or anesthetized. For irradiation, pie cages were placed atop a stack of 4 inches of polystyrene block to prevent back scatter. The total radiation field size allowed two adjacent pie cages to be exposed simultaneously. To verify the uniformity of radiation across all compartments of the two pie cages, radiation dosimetry was carried out for all pie compartments using clinical-grade protocols. Measurements were made with a 0.6 cc Farmer ion chamber (PTW model N30006) with build-up cap, with calibration traceable to the National Institute of Standards and Technology. The dose variation between individual pie compartments was <±2.1%, and the dose variation within individual pie compartments was <±1.8%. From measurements made in a water-filled pie compartment, the estimated dose fall-off from top to bottom of an animal was <2%. The average dose rate across the radiation field was 31.8 cGy/minute reducing to 27 cGy/minute over the duration of the study; mean dose rate for the whole study was therefore 29.7 cGy/minute. Precise exposure time for each radiation dose was calculated on every treatment day taking into account the half-life of ^60^Co. Exposure times varied from 1.7 minutes on average for a 0.5 Gy dose, to an average of 33.7 minutes for a 10 Gy dose.

Three classes of radiation treatment were used for this study: specific treatment doses, sham-irradiated (0 Gy), and naïve (0 Gy). For the specific dose treatment and sham-irradiated treatment groups, each mouse was placed in an individual compartment within the pie cage. Nestlets (Ancare, Bellmore, NY) were included in each compartment of the pie cage for environmental enrichment. Mice were continuously monitored by closed circuit television and demonstrated no visible stress throughout the procedure. Specific radiation doses and the sham and naïve controls were scheduled according to a randomized matrix. The radiation doses administered were 0.5, 1, 1.5, 2, 2.5, 3, 4, 6, 8, or 10 Gy. The number of mice in each dose group and time point that were included in the final data set is provided in Table 1.

Mice exposed to specific radiation doses and the sham-irradiated mice were treated identically except that the ^60^Co source remained shielded during the sham treatments (0 Gy). The treatment times for mice in the sham-irradiated group were matched to the exposure time for the highest specific radiation dose being administered on a given day. Typically, two pie cages each containing 10 mice were exposed simultaneously to either a specific treatment dose or 0 Gy during one dosing session. Three to 6 dosing sessions comprising a total of 60 to 120 mice were performed per week. After treatment, mice were returned to their normal housing cages. Mice in the groups that were naïve to radiation remained in their normal housing environment and were not placed into pie cages or transported to the radiation facility. Transportation of mice occurred by automobile and the animals were kept in insulated containers to maintain the air temperature at approximately 21°C. The only differences with respect to treatment of the sham and naïve animals were the acts of inserting the sham mice into the pie cages and transporting them to and from the radiation facility, a distance of 1 km in each direction.

### Blood collection and preservation

Blood was collected from mice in every treatment group at one of six time points: 0.5, 1, 2, 3, 5, or 7 days after radiation. Blood was collected under CO_2_-induced narcosis by cardiac puncture using heparinized 3 cc syringes, then stabilized with PAXgene Blood RNA stabilization solution (PreAnalyitX GmbH, Hombrechtikon, Switzerland) at a ratio of 1 mL blood to 2.76 mL PAXgene solution. Tubes containing stabilized blood were gently rocked for 24 hours at room temperature and stored at -20°C until RNA isolation.

### RNA isolation and cDNA synthesis

Frozen PAXgene stabilized blood samples were thawed to room temperature and RNA was isolated using PAXgene 96 Blood RNA kits for the purification of cellular RNA according to the manufacturer’s instructions. Nucleic acid from each sample was quantified spectrophotometrically. Isolated RNA was stored at -80°C, then cDNA was synthesized from RNA using Ready-To-Go You-Prime First-Strand Beads (GE Healthcare, Piscataway, NJ) according to the manufacturer’s instructions using random hexamer DNA primers (Integrated DNA Technologies, Coralville, IA). cDNA was stored at -20°C until analyzed by real time quantitative PCR (qPCR).

### Real time quantitative PCR

cDNA synthesized from PAXgene-preserved mouse blood samples was analyzed by qPCR using custom TaqMan® low density arrays (TLDA) in a 384-well microfluidic card in a 4-sample by 96-gene format, or an 8-sample by 48-gene format (Applied Biosystems, Foster City, CA) following the manufacturer’s instructions. Over the course of this experiment, 3 different TLDA configurations were used. The TaqMan^®^ assays on the initial TLDA configuration were selected from a variety of sources, including [18,19] as well as our own published [20] human gene expression data. An interim data analysis indicated that several TaqMan^®^ assays on the initial TLDA configuration did not provide useful data. These assays were removed from the subsequent TLDA configurations. The final TLDA configuration contained 48 TaqMan^®^ assays corresponding to genes that were radiation-responsive or had stable expression across radiation doses, the latter being candidates for endogenous control (EC) genes. A list of gene symbols and names corresponding to the TaqMan® assays used in this study and presented in this paper is shown in Table 8; a list of all 106 TaqMan® assays evaluated in this study is provided in Table S5 in File S1.

**Table 8:** Targeted RNA sequences: corresponding gene names and their TaqMan® assay numbers.

**Gene symbol for targeted RNA sequence**	**Gene name**	**TaqMan® assay**
Actb	actin, beta	Mm00607939_s1
B2m	beta-2 microglobulin	Mm00437764_m1
Bad	BCL2-associated agonist of cell death	Mm00432042_m1
Bax	BCL2-associated X protein	Mm00432051_m1
Bbc3	BCL2 binding component 3	Mm00519268_m1
Brca1	breast cancer 1	Mm01249844_m1
Ccna2	cyclin A2	Mm00438064_m1
Ccng1	cyclin G1	Mm00438084_m1
Cdkn1a	cyclin-dependent kinase inhibitor 1A (P21)	Mm00432448_m1
Erp44	endoplasmic reticulum protein 44	Mm00466483_m1
Fuca1	fucosidase, alpha-L- 1, tissue	Mm00502778_m1
Gtf3a	general transcription factor III A	Mm00550608_m1
Hdac2	histone deacetylase 2	Mm00515108_m1
Hmbs	hydroxymethylbilane synthase	Mm01143545_m1
Hprt1	hypoxanthine phosphoribosyltransferase 1	Mm03024075_m1
Plxnb2	plexin B2	Mm00507118_m1
Prkdc	protein kinase, DNA activated, catalytic polypeptide	Mm01342967_m1
Rb1	retinoblastoma 1	Mm00485586_m1
Ssr1	signal sequence receptor, alpha	Mm00503135_m1
Stom	stomatin	Mm00469130_m1
Ticam2	toll-like receptor adaptor molecule 2	Mm01260003_m1
Tlk1	tousled-like kinase 1	Mm00554286_m1
Tmem168	transmembrane protein 168	Mm00551402_m1

All qPCR assays were run on a 7900HT Real-Time PCR System (Applied Biosystems). Approximately 1.2 ng of mouse cDNA was applied to each well of the TLDA plate. After PCR amplification, the data obtained from an individual well on the 384-well microfluidic card were omitted from further analysis if that well was flagged by the default settings of the SDS version 2.3 software (Applied Biosystems). The SDS flags employed were: Bad Passive Reference, Empty Well, Has Noise Spikes, High Relative Noise, and Non-Amplified Well. If a single mouse had ≥ 1/3 of the TaqMan® assays flagged and omitted, then the entire mouse was omitted from the study.

Cycle threshold (Ct) values were calculated by the software QPCR version 0.9.12 [21] using the AnalyzerMiner algorithm [22]. Ct values were exported from QPCR for further analyses. RNA sequences used as EC genes were chosen based on two criteria: (1) the maximum stability of the Ct value for the respective TaqMan® assay across the entire study, and (2) the Ct value for the chosen TaqMan® assay had to be valid in at least 93% of the mice. The GeNorm algorithm [23] provided the basis for the endogenous control gene selection process in the DataAssist software version 2.0 (Applied Biosystems). Stability for candidate EC genes was assessed by computing the standard deviation of Ct values across doses, and a slope for a regression of its raw Ct values versus dose. For genes with the lowest standard deviations and slopes, M-values (stability factor) were computed to assess their consistency, and were determined from the 28 assays having valid data from over 95% of the samples by using DataAssist which employs the algorithm described by Vandesompele et al. [24]. The assays processed by DataAssist were ranked by M-value and approximately half the assays with the lowest M-value were reprocessed by DataAssist to calculate new M-values. Four rounds of these analyses were performed, reducing to 3 the number of assays which were then selected as the EC genes (Table 2).

### Statistical analyses - variable selection

A variation of forward variable selection was used to choose genes for further analysis. A single set of variables, where each variable corresponds to a transcript for a specific gene, was selected for each pair of adjacent experimental time points, i.e., 0.5 days and 1 day, 1 and 2 days, 2 and 3 days, 3 and 5 days, and 5 and 7 days. These sets of variables are referred to as gene sets A, B, C, D, and E, respectively. Basing the models at adjacent time points on common gene sets allows for the potential prediction of dosimetry between the specific time points evaluated here.

The following criteria were used to identify the best candidate variable (i.e. gene product) for inclusion in each model. First, the total R^2^ for the model including the potential new variable was required to increase by at least 0.01 (i.e. 1% additional variance explained) compared to the model without that potential new variable. Second, the total sample size with the potential variable added was required to be at least 85% of the maximum available. Third, the p-value for the potential variable in the new model was required to be <0.05. One and two degree of freedom p-values were used for linear and quadratic polynomial fits, respectively, for each potential variable. Fourth, the potential variable had to be among the best for each pair of adjacent experimental time points, and to have the best average of the average percentages of the maximum sample size and R^2^ at a particular step, as explained below. That is, the total R^2^ for a model with a potential new variable was expressed as a percentage of the model with the highest R^2^ at a particular step. Similarly the total sample size for a model with a potential new variable was expressed as a percentage of the sample size for the model with the highest available sample size at a particular step. These two percentages were averaged, and those average percentages from the two adjacent time points being considered were again averaged, and the gene product with the best average of the averages was added to the model provided it met all the criteria indicated above.

### Statistical analyses - further variable assessment

Given the very restrictive nature of the above variable selection process, further provisional models were assessed even if they did not meet all the restrictions. That is, the stepwise variable selection process was continued to give expanded “provisional” multiple regression models that were used to evaluate where unselected variables might come out in the process.

### Statistical analyses – cross validation

 Model reliability was assessed using cross-validation, where the total sample was split into halves (“Split 1” and “Split 2”). The model building process was used on each half, and then the models derived in each case were fit to the other half of the data; i.e. variables and coefficients derived on Split 1 were applied to the Split 2 dataset, and vice versa. The R-squared statistics and areas under the ROC curves were computed and then compared along with variables selected in each case.

## Supporting Information

Figure S1 **Relationship between the actual dose administered and the dose predicted by each model.** The gene transcripts used in these models are provided in Tables 4, 5, and 6. Lines and symbols are as described in Figure 2.
(TIF)

Figure S2 **Relationship between the actual dose administered and the dose predicted by each model.** The gene transcripts used in these models are provided in Tables 6 and 7. Lines and symbols are as described in Figure 2.
(TIF)

Figure S3 **Receiver-operator characteristic curves.** Large areas under the curve, as shown here, indicate high levels of sensitivity and specificity of the models. These curves correspond to the models shown in Figure S1.
(TIF)

Figure S4 **Receiver-operator characteristic curves.** Large areas under the curve, as shown here, indicate high levels of sensitivity and specificity of the models. These curves correspond to the models shown in Figure S2.
(TIF)

File S1 **Supporting Tables**. Tables S1a to S1e. Stepwise variable selection summaries for Subsample 1 of the data. Shown are the results for Days 0.5 and 1, Days 1 and 2, Days 2 and 3, Days 3 and 5, and Days 5 and 7. Each table indicates the number of steps in each stepwise analysis, the new variable(s) added at each step, and for each pair of days the amount of variance (R^2^) explained by the model and the corresponding sample size (N). Tables S2a to S2e. Stepwise variable selection summaries for Subsample 2 of the data. Shown are the results for Days 0.5 and 1, Days 1 and 2, Days 2 and 3, Days 3 and 5, and Days 5 and 7. Each table indicates the number of steps in each stepwise analysis, the new variable(s) added at each step, and for each pair of days the amount of variance (R^2^) explained by the model and the corresponding sample size (N). Table S3. Cross validation results with split samples for Split 1. Shown for each pair of days and each combination of split analyses are the predicted R^2^ values, the change in R^2^, the area under the receiver-operator characteristic (ROC) curve, and the change in the area under the curve. Table S4. Cross validation results with split samples for Split 2. Shown for each pair of days and each combination of split analyses are the predicted R^2^ values, the change in R^2^, the area under the receiver-operator characteristic (ROC) curve, and the change in the area under the curve. Table S5. Targeted RNA sequences: corresponding gene names and their TaqMan® assay numbers. Shown are the gene symbol, gene name, and the TaqMan® assay numbers for each of the RNA sequences analyzed in this study.
(DOCX)
