# Supplementary figures and images for: Gene Expression-Based Dosimetry by Dose and Time in Mice Following Acute Radiation Exposure
Source: PLoS One. 2013 Dec 16;8(12):e83390. doi: 10.1371/journal.pone.0083390 (PMC3865163; doi:10.1371/journal.pone.0083390)

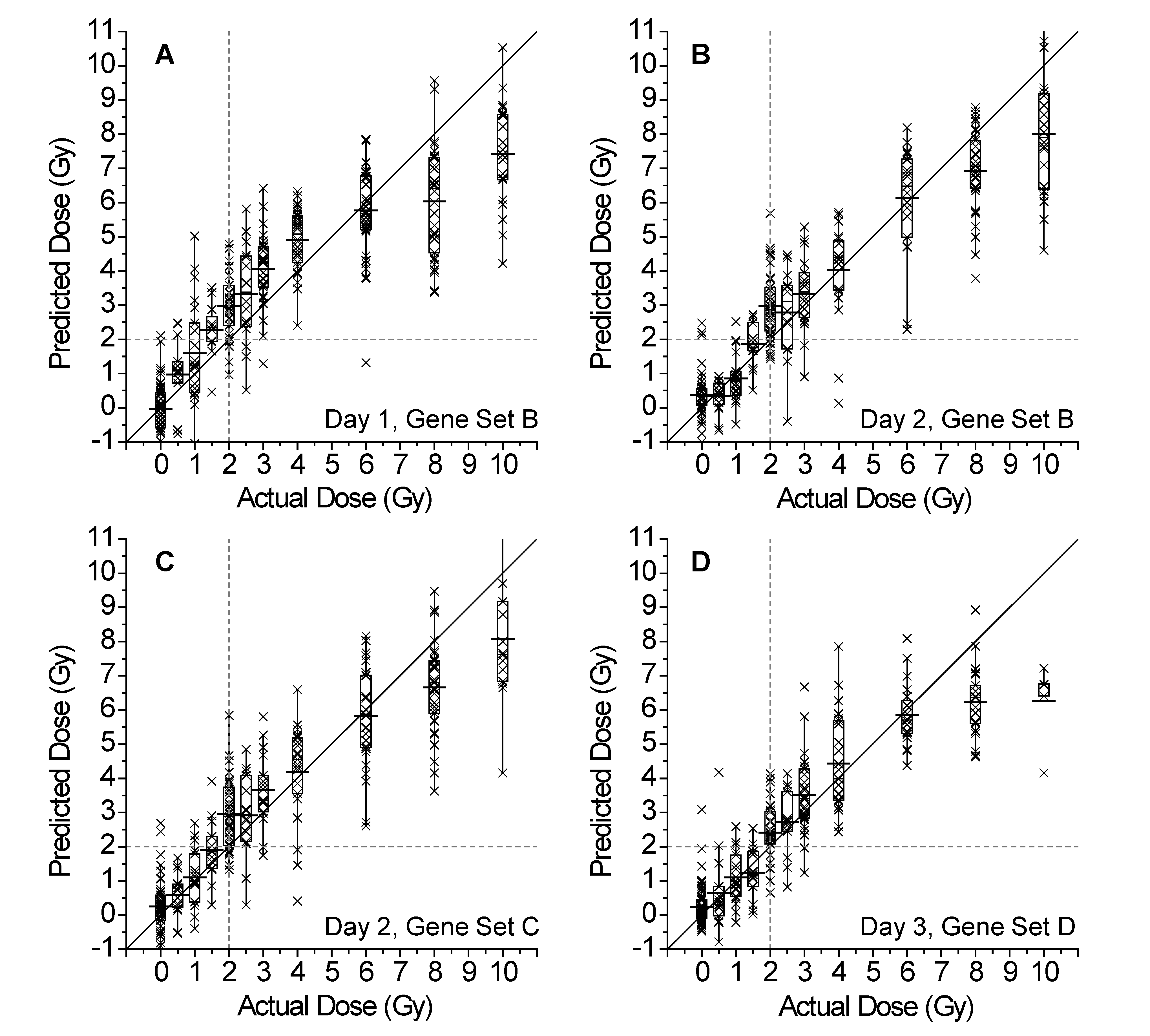

Supplement: Figure S1 — Relationship between the actual dose administered and the dose predicted by each model. The gene transcripts used in these models are provided in Tables 4, 5, and 6. Lines and symbols are as described in Figure 2. (TIF) [file pone.0083390.s001.tif]

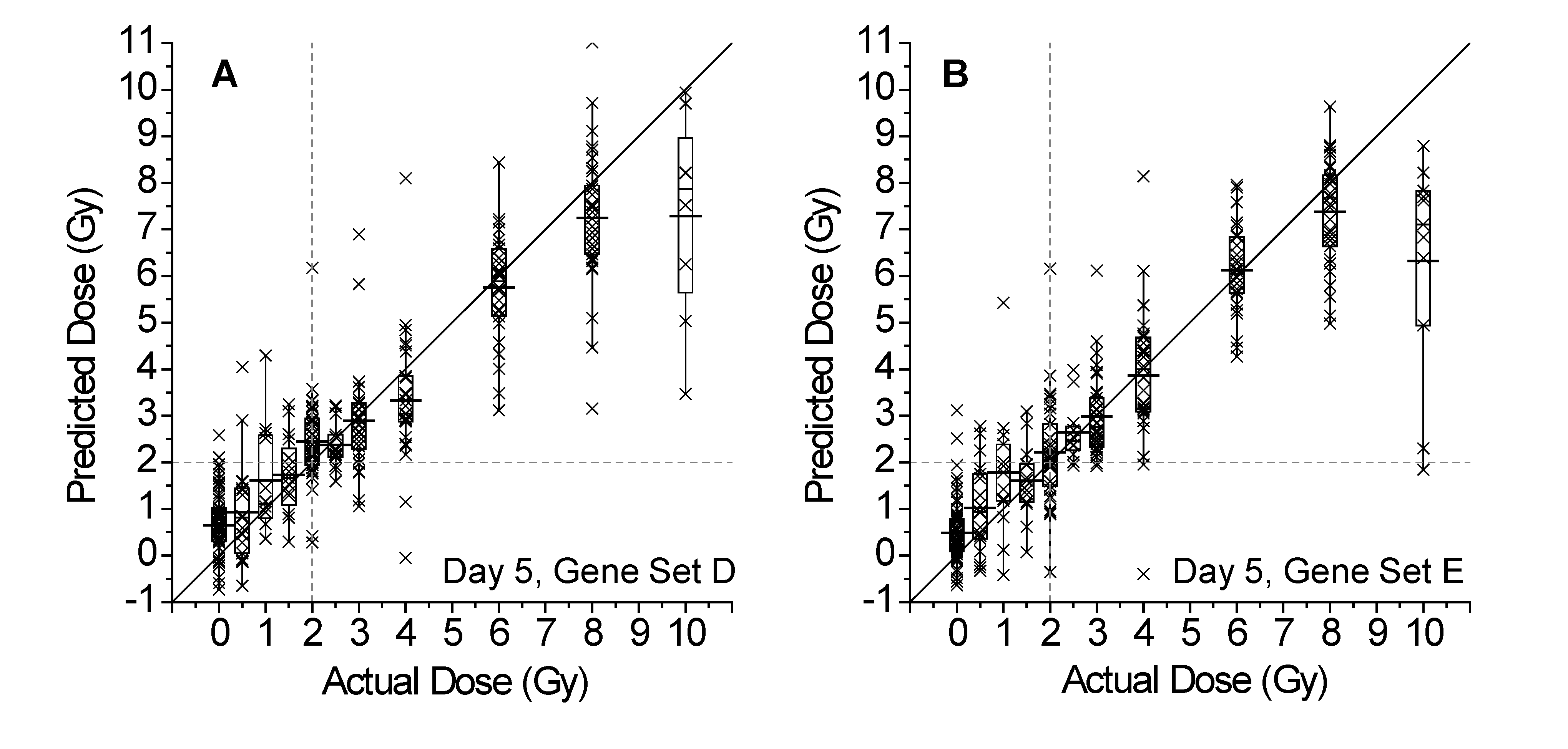

Supplement: Figure S2 — Relationship between the actual dose administered and the dose predicted by each model. The gene transcripts used in these models are provided in Tables 6 and 7. Lines and symbols are as described in Figure 2. (TIF) [file pone.0083390.s002.tif]
