# Supplementary material for: Gene Expression-Based Dosimetry by Dose and Time in Mice Following Acute Radiation Exposure
Source: PLoS One. 2013 Dec 16;8(12):e83390. doi: 10.1371/journal.pone.0083390 (PMC3865163; doi:10.1371/journal.pone.0083390)
Supplement: File S1 — Supporting Tables. Tables S1a to S1e. Stepwise variable selection summaries for Subsample 1 of the data. Shown are the results for Days 0.5 and 1, Days 1 and 2, Days 2 and 3, Days 3 and 5, and Days 5 and 7. Each table indicates the number of steps in each stepwise analysis, the new variable(s) added at each step, and for each pair of days the amount of variance (R2) explained by the model and the corresponding sample size (N). Tables S2a to S2e. Stepwise variable selection summaries for Subsample 2 of the data. Shown are the results for Days 0.5 and 1, Days 1 and 2, Days 2 and 3, Days 3 and 5, and Days 5 and 7. Each table indicates the number of steps in each stepwise analysis, the new variable(s) added at each step, and for each pair of days the amount of variance (R2) explained by the model and the corresponding sample size (N). Table S3. Cross validation results with split samples for Split 1. Shown for each pair of days and each combination of split analyses are the predicted R2 values, the change in R2, the area under the receiver-operator characteristic (ROC) curve, and the change in the area under the curve. Table S4. Cross validation results with split samples for Split 2. Shown for each pair of days and each combination of split analyses are the predicted R2 values, the change in R2, the area under the receiver-operator characteristic (ROC) curve, and the change in the area under the curve. Table S5. Targeted RNA sequences: corresponding gene names and their TaqMan® assay numbers. Shown are the gene symbol, gene name, and the TaqMan® assay numbers for each of the RNA sequences analyzed in this study. (DOCX) [file pone.0083390.s005.docx]

Tables S1a to S1e are for Subsample 1 of the data.

Table S1a. Stepwise variable selection summary for days 0.5 and 1

|  |  | **Day 0.5 (N=112)** | | **Day 1 (N=191)** | |
| --- | --- | --- | --- | --- | --- |
| **Step** | **(New) Variable(s)** | **R^2^** | **N (% Max)** | **R^2^** | **N (% Max)** |
| 1 | Cdkn1a, Cdkn1a_sq | 0.450 | 106 (94.6%) | 0.602 | 185 (96.9%) |
| 2 | Ticam2, Ticam2_sq | 0.644 | 103 (92.0%) | 0.649 | 173 (90.6%) |
| 3 | Rb1 | 0.659 | 103 (92.0%) | 0.685 | 173 (90.6%) |
| 4 | Prkdc, Prkdc_sq | 0.728 | 100 (89.4%) | 0.721 | 163 (85.3%) |
| 5 | Tlk1, Tlk1_sq | 0.756 | 98 (87.5%) | 0.739 | 163 (85.3%) |

Table S1b. Stepwise variable selection summary for days 1 and 2

|  |  | **Day 1 (N=191)** | | **Day 2 (N=136)** | |
| --- | --- | --- | --- | --- | --- |
| **Step** | **(New) Variable(s)** | **R^2^** | **N (% Max)** | **R^2^** | **N (% Max)** |
| 1 | Cdkn1a, Cdkn1a_sq | 0.602 | 185 (96.9%) | 0.588 | 129 (94.9%) |
| 2 | Rb1, Rb1_sq | 0.639 | 185 (96.9%) | 0.721 | 127 (93.4%) |
| 3 | Tlk1 | 0.686 | 185 (96.9%) | 0.761 | 124 (91.2%) |
| 4 | Fuca1, Fuca1_sq | 0.697 | 185 (96.9%) | 0.777 | 124 (91.2%) |
| 5 | Apc, Apc_sq | 0.717 | 183 (95.8%) | 0.789 | 121 (89.0%) |
| 6 | Ccng1, Ccng1_sq | 0.737 | 181 (94.8%) | 0.800 | 118 (86.8%) |
| 7 | Ccna2, Ccna2_sq | 0.752 | 181 (94.8%) | 0.818 | 116 (85.3%) |

Table S1c. Stepwise variable selection summary for days 2 and 3

|  |  | **Day 2 (N=136)** | | **Day 3 (N=166)** | |
| --- | --- | --- | --- | --- | --- |
| **Step** | **(New) Variable(s)** | **R^2^** | **N (% Max)** | **R^2^** | **N (% Max)** |
| 1 | Cdkn1a, Cdkn1a_sq | 0.588 | 129 (94.9%) | 0.582 | 161 (97.0%) |
| 2 | Rb1, Rb1_sq | 0.721 | 127 (93.4%) | 0.683 | 161 (97.0%) |
| 3 | Ccng1, Ccng1_sq | 0.743 | 123 (90.4%) | 0.716 | 160 (96.4%) |
| 4 | Ccna2, Ccna2_sq | 0.767 | 121 (89.0%) | 0.740 | 156 (94.0%) |
| 5 | Bad, Bad_sq | 0.793 | 119 (87.5%) | 0.756 | 154 (92.8%) |

Table S1d. Stepwise variable selection summary for days 3 and 5

|  |  | **Day 3 (N=166)** | | **Day 5 (N=163)** | |
| --- | --- | --- | --- | --- | --- |
| **Step** | **(New) Variable(s)** | **R^2^** | **N (% Max)** | **R^2^** | **N (% Max)** |
| 1 | Cdkn1a, Cdkn1a_sq | 0.582 | 161 (97.0%) | 0.620 | 159 (98.1%) |
| 2 | Rb1, Rb1_sq | 0.683 | 160 (96.4%) | 0.756 | 159 (98.1%) |
| 3 | Ccng1, Ccng1_sq | 0.716 | 160 (96.4%) | 0.778 | 155 (95.7%) |
| 4 | Gadd45a, Gadd45a_sq | 0.742 | 158 (95.2%) | 0.800 | 154 (95.1%) |
| 5 | Ddb1, Ddb1_sq | 0.787 | 154 (92.8%) | 0.810 | 151 (93.1%) |
| 6 | Tfrc | 0.790 | 154 (92.8%) | 0.821 | 149 (92.0%) |

Table S1e. Stepwise variable selection summary for days 5 and 7

|  |  | **Day 5 (N=163)** | | **Day 7 (N=130)** | |
| --- | --- | --- | --- | --- | --- |
| **Step** | **(New) Variable(s)** | **R^2^** | **N (% Max)** | **R^2^** | **N (% Max)** |
| 1 | Cdkn1a, Cdkn1a_sq | 0.620 | 159 (98.1%) | 0.518 | 125 (96.2%) |
| 2 | Ccna2, Ccna2_sq | 0.778 | 157 (96.9%) | 0.635 | 123 (94.6%) |
| 3 | Ticam2 | 0.834 | 148 (91.4%) | 0.714 | 120 (92.3%) |
| 4 | Trfc | 0.849 | 145 (89.5%) | 0.724 | 116 (89.2%) |

Tables S2a to S2e are for Subsample 2 of the data.

Table S2a. Stepwise variable selection summary for days 0.5 and 1

|  |  | **Day 0.5 (N=99)** | | **Day 1 (N=161)** | |
| --- | --- | --- | --- | --- | --- |
| **Step** | **(New) Variable(s)** | **R^2^** | **N (% Max)** | **R^2^** | **N (% Max)** |
| 1 | Cdkn1a, Cdkn1a_sq | 0.571 | 97 (98.0%) | 0.551 | 152 (94.4%) |
| 2 | Plnxb2, Plnxb2_sq | 0.655 | 95 (96.0%) | 0.585 | 150 (93.2%) |
| 3 | Cd164 | 0.675 | 91 (91.9%) | 0.607 | 149 (92.5%) |
| 4 | Stom, Stom_sq | 0.685 | 91 (91.9%) | 0.658 | 144 (89.4%) |
| 5 | Hmbs | 0.718 | 90 (90.9%) | 0.706 | 137 (85.1%) |
| 6 | Fuca1, Fuca1_sq | 0.730 | 90 (90.9%) | 0.716 | 137 (85.1%) |

Table S2b. Stepwise variable selection summary for days 1 and 2

|  |  | **Day 1 (N=161)** | | **Day 2 (N=148)** | |
| --- | --- | --- | --- | --- | --- |
| **Step** | **(New) Variable(s)** | **R^2^** | **N (% Max)** | **R^2^** | **N (% Max)** |
| 1 | Cdkn1a, Cdkn1a_sq | 0.551 | 152 (94.4%) | 0.657 | 143 (96.6%) |
| 2 | Rb1, **Rb1_sq** | 0.592 | 150 (93.2%) | 0.713 | 143 (96.6%) |
| 3 | Tlk1 | 0.627 | 149 (92.5%) | 0.757 | 139 (93.9%) |
| 4 | Prdx4, Prdx4_sq | 0.640 | 149 (92.5%) | 0.781 | 139 (93.9%) |
| 5 | Fuca1, Fuca1_sq | 0.654 | 148 (91.9%) | 0.807 | 139 (93.9%) |
| 6 | Ppib | 0.680 | 146 (90.7%) | 0.823 | 136 (91.9%) |

Table S2c. Stepwise variable selection summary for days 2 and 3

|  |  | **Day 2 (N=148)** | | **Day 3 (N=140)** | |
| --- | --- | --- | --- | --- | --- |
| **Step** | **(New) Variable(s)** | **R^2^** | **N (% Max)** | **R^2^** | **N (% Max)** |
| 1 | Cdkn1a, Cdkn1a_sq | 0.657 | 143 (96.6%) | 0.462 | 134 (95.7%) |
| 2 | Rb1, Rb1_sq | 0.713 | 143 (96.6%) | 0.713 | 133 (95.0%) |
| 3 | Fuca1, Fuca1_sq | 0.752 | 142 (95.9%) | 0.695 | 133 (95.0%) |
| 4 | Prdx4, Prdx4_sq | 0.774 | 142 (95.9%) | 0.707 | 133 (95.0%) |
| 5 | Hdac | 0.798 | 136 (91.9%) | 0.783 | 129 (92.1%) |
| 6 | Trfc | 0.829 | 133 (89.9%) | 0.793 | 127 (90.7%) |
| 7 | Ticam2 | 0.839 | 130 (87.8%) | 0.829 | 123 (87.9%) |

Table S2d. Stepwise variable selection summary for days 3 and 5

|  |  | **Day 3 (N=140)** | | **Day 5 (N=162)** | |
| --- | --- | --- | --- | --- | --- |
| **Step** | **(New) Variable(s)** | **R^2^** | **N (% Max)** | **R^2^** | **N (% Max)** |
| 1 | Ccna2, Ccna2_sq | 0.549 | 134 (95.7%) | 0.493 | 159 (96.9%) |
| 2 | Cdkn1a, Cdkn1a_sq | 0.725 | 130 (92.9%) | 0.688 | 152 (93.8%) |
| 3 | Gadd45a | 0.735 | 129 (91.4%) | 0.701 | 151 (93.2%) |
| 4 | Ticam2 | 0.745 | 124 (88.6%) | 0.758 | 141 (87.0%) |
| 5 | Dpm1 | 0.779 | 121 (86.4%) | 0.771 | 139 (85.8%) |

Table S2e. Stepwise variable selection summary for days 5 and 7

|  |  | **Day 5 (N=162)** | | **Day 7 (N=186)** | |
| --- | --- | --- | --- | --- | --- |
| **Step** | **(New) Variable(s)** | **R^2^** | **N (% Max)** | **R^2^** | **N (% Max)** |
| 1 | Cdkn1a, Cdkn1a_sq | 0.495 | 156 (96.3%) | 0.553 | 171 (91.9%) |
| 2 | Rb1, Rb1_sq | 0.673 | 155 (95.7%) | 0.664 | 169 (90.9%) |
| 3 | Ticam2 | 0.743 | 145 (89.5%) | 0.704 | 166 (89.2%) |
| 4 | Ccna2, Ccna2_sq | 0.755 | 141 (87.0%) | 0.714 | 166 (89.2%) |

Table S3. Cross validation results with split samples

| **Days used for Model Estimation** | **Day’s Data Fit** | **Estimate is made from** | **Fit is made on** | **R^2^_prediction_** | **Change in R^2^** | **Area Under ROC curve** | **Change in Area** |
| --- | --- | --- | --- | --- | --- | --- | --- |
| 0.5 & 1 | 0.5 | split 1 | split 1 | 0.756 |  | 0.957 |  |
| 0.5 & 1 | 0.5 | split 1 | split 2 | 0.497 | -0.258 | 0.900 | -0.057 |
| 0.5 & 1 | 1 | split 1 | split 1 | 0.739 |  | 0.973 |  |
| 0.5 & 1 | 1 | split 1 | split 2 | 0.644 | -0.094 | 0.976 | 0.003 |
| 1 & 2 | 1 | split 1 | split 1 | 0.752 |  | 0.972 |  |
| 1 & 2 | 1 | split 1 | split 2 | 0.656 | -0.096 | 0.974 | 0.002 |
| 1 & 2 | 2 | split 1 | split 1 | 0.818 |  | 0.952 |  |
| 1 & 2 | 2 | split 1 | split 2 | 0.708 | -0.109 | 0.941 | -0.011 |
| 2 & 3 | 2 | split 1 | split 1 | 0.793 |  | 0.971 |  |
| 2 & 3 | 2 | split 1 | split 2 | 0.633 | -0.159 | 0.943 | -0.028 |
| 2 & 3 | 3 | split 1 | split 1 | 0.756 |  | 0.987 |  |
| 2 & 3 | 3 | split 1 | split 2 | 0.646 | -0.110 | 0.927 | -0.060 |
| 3 & 5 | 3 | split 1 | split 1 | 0.786 |  | 0.990 |  |
| 3 & 5 | 3 | split 1 | split 2 | 0.543 | -0.338 | 0.928 | -0.062 |
| 3 & 5 | 5 | split 1 | split 1 | 0.802 |  | 0.959 |  |
| 3 & 5 | 5 | split 1 | split 2 | 0.674 | -0.098 | 0.917 | -0.042 |
| 5 & 7 | 5 | split 1 | split 1 | 0.849 |  | 0.949 |  |
| 5 & 7 | 5 | split 1 | split 2 | 0.698 | -0.151 | 0.919 | -0.030 |
| 5 & 7 | 7 | split 1 | split 1 | 0.724 |  | 0.931 |  |
| 5 & 7 | 7 | split 1 | split 2 | 0.601 | -0.123 | 0.865 | -0.066 |

Table S4. Cross validation results with split samples

| **Days used for Model Estimation** | **Day’s Data Fit** | **Estimate is made from** | **Fit is made on** | **R^2^_prediction_** | **Change in R^2^** | **Area Under ROC curve** | **Change in Area** |
| --- | --- | --- | --- | --- | --- | --- | --- |
| 0.5 & 1 | 0.5 | split 2 | split 2 | 0.730 |  | 0.943 |  |
| 0.5 & 1 | 0.5 | split 2 | split 1 | 0.352 | -0.378 | 0.929 | -0.014 |
| 0.5 & 1 | 1 | split 2 | split 2 | 0.716 |  | 0.969 |  |
| 0.5 & 1 | 1 | split 2 | split 1 | 0.685 | -0.031 | 0.961 | -0.008 |
| 1 & 2 | 1 | split 2 | split 2 | 0.680 |  | 0.981 |  |
| 1 & 2 | 1 | split 2 | split 1 | 0.681 | 0.001 | 0.975 | -0.006 |
| 1 & 2 | 2 | split 2 | split 2 | 0.822 |  | 0.971 |  |
| 1 & 2 | 2 | split 2 | split 1 | 0.699 | -0.123 | 0.960 | -0.011 |
| 2 & 3 | 2 | split 2 | split 2 | 0.839 |  | 0.969 |  |
| 2 & 3 | 2 | split 2 | split 1 | 0.686 | -0.153 | 0.946 | -0.023 |
| 2 & 3 | 3 | split 2 | split 2 | 0.819 |  | 0.969 |  |
| 2 & 3 | 3 | split 2 | split 1 | 0.635 | -0.184 | 0.964 | -0.005 |
| 3 & 5 | 3 | split 2 | split 2 | 0.779 |  | 0.973 |  |
| 3 & 5 | 3 | split 2 | split 1 | 0.637 | -0.143 | 0.985 | 0.012 |
| 3 & 5 | 5 | split 2 | split 2 | 0.771 |  | 0.940 |  |
| 3 & 5 | 5 | split 2 | split 1 | 0.774 | 0.003 | 0.900 | -0.040 |
| 5 & 7 | 5 | split 2 | split 2 | 0.755 |  | 0.924 |  |
| 5 & 7 | 5 | split 2 | split 1 | 0.804 | 0.049 | 0.936 | 0.012 |
| 5 & 7 | 7 | split 2 | split 2 | 0.714 |  | 0.915 |  |
| 5 & 7 | 7 | split 2 | split 1 | 0.664 | -0.051 | 0.951 | 0.036 |

Table S5. Targeted RNA sequences: corresponding gene names and their TaqMan® assay numbers.

| **Gene symbol for targeted RNA sequence** | **Gene name** | **TaqMan® assay** |
| --- | --- | --- |
| 18S | Eukaryotic 18S rRNA | Hs99999901_s1 |
| Abca1 | ATP-binding cassette, sub-family A (ABC1), member 1 | Mm00442663_m1 |
| Actb | actin, beta | Mm00607939_s1 |
| Ada | adenosine deaminase | Mm00545720_m1 |
| Alcam | activated leukocyte cell adhesion molecule | Mm00711623_m1 |
| Alg5 | asparagine-linked glycosylation 5 homolog (yeast, dolichyl-phosphate beta-glucosyltransferase) | Mm00508324_m1 |
| Apc | adenomatosis polyposis coli | Mm00545877_m1 |
| Astn2 | astrotactin 2 | Mm00517123_m1 |
| Atf3 | activating transcription factor 3 | Mm00476032_m1 |
| Atm | ataxia telangiectasia mutated homolog (human) | Mm00431867_m1 |
| Atp1b3 | ATPase, Na+/K+ transporting, beta 3 polypeptide | Mm00783499_s1 |
| Atr | ataxia telangiectasia and Rad3 related | Mm01223626_m1 |
| B2m | beta-2 microglobulin | Mm00437764_m1 |
| B2m | beta-2 microglobulin | Mm00437762_m1 |
| B2m | beta-2 microglobulin | Mm01269327_g1 |
| Bad | BCL2-associated agonist of cell death | Mm00432042_m1 |
| Bax | BCL2-associated X protein | Mm00432051_m1 |
| Bbc3 | BCL2 binding component 3 | Mm00519268_m1 |
| Brca1 | breast cancer 1 | Mm01249844_m1 |
| Ccna2 | cyclin A2 | Mm00438064_m1 |
| Ccng1 | cyclin G1 | Mm00438084_m1 |
| Cd164 | CD164 antigen | Mm00489798_m1 |
| Cdkn1a | cyclin-dependent kinase inhibitor 1A (P21) | Mm00432448_m1 |
| Chaf1a | chromatin assembly factor 1, subunit A (p150) | Mm00516472_m1 |
| Chek1 | checkpoint kinase 1 homolog (S. pombe) | Mm00432485_m1 |
| Cxcl10 | chemokine (C-X-C motif) ligand 10 | Mm99999072_m1 |
| Ddb1 | damage specific DNA binding protein 1 | Mm00497159_m1 |
| Ddb2 | damage specific DNA binding protein 2 | Mm00472176_m1 |
| Dpm1 | dolichol-phosphate (beta-D) mannosyltransferase 1 | Mm00494520_m1 |
| E2f1 | E2F transcription factor 1 | Mm00432939_m1 |
| Entpd1 | ectonucleoside triphosphate diphosphohydrolase 1 | Mm00515447_m1 |
| Erp44 | endoplasmic reticulum protein 44 | Mm00466483_m1 |
| Exo1 | exonuclease 1 | Mm00516302_m1 |
| Fancc | Fanconi anemia, complementation group C | Mm00514846_m1 |
| Fas | Fas (TNF receptor superfamily member 6) | Mm00433237_m1 |
| Fuca1 | fucosidase, alpha-L- 1, tissue | Mm00502778_m1 |
| Gadd45a | growth arrest and DNA-damage-inducible 45 alpha | Mm00432802_m1 |
| Gapdh | glyceraldehyde-3-phosphate dehydrogenase | Mm99999915_g1 |
| Gapdh;ENSMUSG00000073212;Gm5944;Gm12070 | glyceraldehyde-3-phosphate dehydrogenase;predicted gene, ENSMUSG00000073212;predicted gene, EG546363;predicted gene 12070 | Mm03302249_g1 |
| Gdf15 | growth differentiation factor 15 | Mm00442228_m1 |
| Gja1 | gap junction protein, alpha 1 | Mm00439105_m1 |
| Glg1 | golgi apparatus protein 1 | Mm00486029_m1 |
| Gtf3a | general transcription factor III A | Mm00550608_m1 |
| Hdac2 | histone deacetylase 2 | Mm00515108_m1 |
| Hmbs | hydroxymethylbilane synthase | Mm01143545_m1 |
| Hprt1 | hypoxanthine guanine phosphoribosyl transferase 1 | Mm03024075_m1 |
| Itfg1 | integrin alpha FG-GAP repeat containing 1 | Mm00472146_m1 |
| Itm2b | integral membrane protein 2B | Mm00515213_m1 |
| Lamp3 | lysosomal-associated membrane protein 3 | Mm00616604_m1 |
| Lig1 | ligase I, DNA, ATP-dependent | Mm00495331_m1 |
| Lipa | lysosomal acid lipase A | Mm00498820_m1 |
| LOC236598 | 28S ribosomal RNA | Mm03682676_s1 |
| Mdm2 | transformed mouse 3T3 cell double minute 2 | Mm01233136_m1 |
| Mlh1 | mutL homolog 1 (E. coli) | Mm00503449_m1 |
| Mlh3 | mutL homolog 3 (E coli) | Mm01302907_m1 |
| Msh2 | mutS homolog 2 (E. coli) | Mm00500567_m1 |
| Msh3 | mutS homolog 3 (E. coli) | Mm00487756_m1 |
| Msh6 | mutS homolog 6 (E. coli) | Mm01227378_m1 |
| Mtap4 | microtubule-associated protein 4 | Mm00485247_m1 |
| Nthl1 | nth (endonuclease III)-like 1 (E.coli) | Mm00476559_m1 |
| Pgk1 | phosphoglycerate kinase 1 | Mm00435617_m1 |
| Plxnb2 | plexin B2 | Mm00507118_m1 |
| Pmaip1 | phorbol-12-myristate-13-acetate-induced protein 1 | Mm00451763_m1 |
| Pms2 | postmeiotic segregation increased 2 (S. cerevisiae) | Mm01200871_m1 |
| Pold1 | polymerase (DNA directed), delta 1, catalytic subunit | Mm00448253_m1 |
| Pold3 | polymerase (DNA-directed), delta 3, accessory subunit | Mm00713051_m1 |
| Ppan | peter pan homolog (Drosophila) | Mm00524057_m1 |
| Ppib | peptidylprolyl isomerase B | Mm00478295_m1 |
| Prdx4 | peroxiredoxin 4 | Mm00450261_m1 |
| Prkdc | protein kinase, DNA activated, catalytic polypeptide | Mm01342967_m1 |
| Ptprk | protein tyrosine phosphatase, receptor type, K | Mm00436070_m1 |
| Rad51c | RAD51 homolog c (S. cerevisiae) | Mm01307097_m1 |
| Rad51c | RAD51 homolog c (S. cerevisiae) | Mm00475154_m1 |
| Rb1 | retinoblastoma 1 | Mm00485586_m1 |
| Rrm2b | ribonucleotide reductase M2 B (TP53 inducible) | Mm01165706_m1 |
| Sdha | succinate dehydrogenase complex, subunit A, flavoprotein (Fp) | Mm01352357_m1 |
| Sec62 | SEC62 homolog (S. cerevisiae) | Mm01289462_m1 |
| Slc25a36 | solute carrier family 25, member 36 | Mm00507200_m1 |
| Slc39a6 | solute carrier family 39 (metal ion transporter), member 6 | Mm00507295_m1 |
| Spcs2 | signal peptidase complex subunit 2 homolog (S. cerevisiae) | Mm00651159_m1 |
| Srd5a2 | steroid 5 alpha-reductase 2 | Mm00446421_m1 |
| Ssr1 | signal sequence receptor, alpha | Mm00503135_m1 |
| Stom | stomatin | Mm00469130_m1 |
| Taf1a | TATA box binding protein (Tbp)-associated factor, RNA polymerase I, A | Mm00498829_m1 |
| Taf7 | TAF7 RNA polymerase II, TATA box binding protein (TBP)-associated factor | Mm00496299_m1 |
| Tbp | TATA box binding protein | Mm01277045_m1 |
| Tert | telomerase reverse transcriptase | Mm00436931_m1 |
| Tfrc | transferrin receptor | Mm00441941_m1 |
| Tgfbr3 | transforming growth factor, beta receptor III | Mm00803538_m1 |
| Ticam2 | toll-like receptor adaptor molecule 2 | Mm01260003_m1 |
| Tlk1 | tousled-like kinase 1 | Mm00554286_m1 |
| Tm9sf2 | transmembrane 9 superfamily member 2 | Mm00446560_m1 |
| Tmed5 | transmembrane emp24 protein transport domain containing 5 | Mm00547008_m1 |
| Tmem168 | transmembrane protein 168 | Mm00551402_m1 |
| Tmem49 | transmembrane protein 49 | Mm00774656_m1 |
| Tnfrsf10b | tumor necrosis factor receptor superfamily, member 10b | Mm00457866_m1 |
| Tomm70a | translocase of outer mitochondrial membrane 70 homolog A (yeast) | Mm00506907_m1 |
| Trp53 | transformation related protein 53 | Mm01731287_m1 |
| Trp53bp1 | transformation related protein 53 binding protein 1 | Mm00658689_m1 |
| Trpc2 | transient receptor potential cation channel, subfamily C, member 2 | Mm00441984_m1 |
| Ube2a | ubiquitin-conjugating enzyme E2A, RAD6 homolog (S. cerevisiae) | Mm00498012_m1 |
| Wrn | Werner syndrome homolog (human) | Mm00449253_m1 |
| Xpc | xeroderma pigmentosum, complementation group C | Mm01183434_m1 |
| Zfp120 | zinc finger protein 120 | Mm00480988_m1 |
| Zmpste24 | zinc metallopeptidase, STE24 homolog (S. cerevisiae) | Mm00554619_m1 |
